# The outcome of patients with ménière’s disease

**DOI:** 10.1016/S1808-8694(15)30078-1

**Published:** 2015-10-19

**Authors:** Adriana Gonzaga Chaves, Letícia Boari, Mário Sérgio Lei Munhoz

**Affiliations:** a M.S. Student - Department of Otorhinolaryngology and Head and Neck Surgery - UNIFESP-EPM, Otorhinolaryngologist; b M.S in Otorhinolaryngologist - Santa Casa de Misericórdia de São Paulo. Fellow in Neurotology - Department of Otorhinolaryngology and Head and Neck Surgery - UNIFESP-EPM; c Adjunct Professor of Otorhinolaryngology and Head of the Neurotology Discipline of the Department of Otorhinolaryngology and Head and Neck Surgery - UNIFESP-EPM. Discipline of Otorhinolaryngology - Department of Otorhinolaryngology and Head and Neck Surgery - Federal University of São Paulo - Paulista School of Medicine UNIFESP-EPM. São Paulo-SP

**Keywords:** ménière’s disease, hydrops, hearing loss, vertigo, tinnitus

## Abstract

Ménière’s disease is a frequent vestibular disease that occurs predominantly in the fourth decade of life. Diagnosis is mostly medical and is based on findings of vertigo, sensorineural hearing loss, tinnitus and aural fullness. **Aim:** To study the clinical findings of Ménière’s disease: age, duration of vertigo, tinnitus, hearing loss and aural fullness, and unilateral or bilateral involvement. **Method:** a retrospective study included 39 patients with a diagnosis of Ménière’s disease confirmed by electrocochleography, who were seen at a neuro-otology referral centre. Patients underwent a clinical examination, audiometry and bilateral transtympanic electrocochleography. Patients were separated into 2 groups: bilateral Ménière’s disease and unilateral Ménière’s disease. **Results:** The mean age was 42.9 years; 72.5% were female. Fluctuation of hearing loss occurred in 54.5% of cases, and 65.7% had frequent attacks of vertigo. Bilateral disease was observed in 33.3%. The onset of the disease was earlier in the bilateral group (33.7 years) compared to the unilateral group (p= 0.0013). Duration of disease, tinnitus, hearing loss and aural fullness were similar between groups. **Conclusion:** Patients with bilateral Ménière’s disease had symptoms earlier than patients with unilateral disease. There was no difference between the groups in duration of disease and associated symptoms.

## INTRODUCTION

Meniere’s disease was described by Prosper Ménière, in 1861. It represents one of the most common vestibular diseases, with a prevalence rate of 46 to 200 cases for every 100 thousand individuals^1^. There is no gender distribution difference and it usually starts on the fourth decade of life^2^.

Lymphatic hydrops (LH) is the pathological substrate of Meniere’s disease, characterized by a distention of the endolymphatic space^3^.

Hydrops is associated with viral or bacterial infectious processes, immunomediated diseases, abnormalities in temporal bone development, genetic predisposition, trauma, otospongiosis, and others^4^, ^5^, ^6^, ^7^, ^8^, ^9^, ^10^.

Meniere’s disease diagnosis is essentially clinical in nature. It is characterized by recurrent and spontaneous vertigo spells, fluctuating sensorineural hearing loss, tinnitus and ear fullness^11^. In 1972, the hearing and balance committee of the American Academy of Otolaryngology and Head and Neck Surgery (AAO-HNS) defined the parameters for the clinical diagnosis of Meniere’s disease. In 1995, the AAO-HNS enhanced these criteria, making them simple and more easily applicable^11^. According to AAO-HNS criteria, individuals with 2 or more spontaneous vertigo spells, lasting for 20 minutes or more, with documented hearing loss in at least one occasion and tinnitus or ear fullness are clinically classified as having Meniere’s disease^11^. Diagnosis certainty is only possible by means of a post mortem study of the temporal bone^12^.

Electrocochleography is the most adequate test to aid in the diagnosis^13^, ^14^. It is based on recording endocochlear potentials, generated at the time of sound stimulus transduction. The most used potentials for this end are the summation potential (SP) and the action potential (AP) ^15^. The SP reflects the activity of hair cells and, consequently, non-linear movements (vibratory asymmetries) of the base membrane^15^, ^16^, ^17^. The AP portrays the summation of numerous action potentials in the neurofibril, which make up the auditory branch of the eighth cranial nerve^15^, ^16^, ^17^.

The highest reliability parameter is the ratio between the summation potential amplitude and the action potential amplitude (SP/AP ratio). In Meniere’s disease, the alterations in the physical properties and mechanisms of the base membrane, caused by a distention in the scala media, cause changes in the electrical responses triggered by sound stimuli. As a result, the SP/AP ratio is altered because of an increase in SP potential^16^.

One of the major difficulties in approaching a patient with Meniere’s disease has been to establish an objective correlation between clinical symptoms, disease progression and electrocochleography. Characteristically speaking, the disease has periods of remission and exacerbation, with varying vertigo spells duration, intensity and frequency. Symptoms may be present together or in isolation, especially in the initial disease stages, delaying diagnosis and proper treatment. On the other hand, on the second decade of progression, vertigo stabilizes, with a sensitive reduction in crises periodicity. In prior investigations, some authors have observed that, the longer the progression time, the higher is the prevalence of bilateral involvement and, consequently, a greater impact on the patient’s life quality^1^. Moreover, progression time seems to be related to symptoms severity, shown by patients with late development who had hearing loss and less intense physical disability when compared to patients with earlier onset.

The goal of the present investigation is to study the progression of Meniere’s disease in patients with clinical diagnosis established and confirmed by transtympanic electrocochleography, in relation to vertigo spells time of progression and other symptoms (tinnitus, ear fullness, hearing loss) and to age range.

## PATIENTS AND METHODS

This is a transversal descriptive retrospective study, which was submitted to the Research Ethics Committee of the UNIFESP/EPM and was approved according to protocol number 1780/06.

54 Meniere’s disease patients were assessed, seen at the reference clinic, from February to November of 2005, according to the clinical criteria that were proposed and revised in 1995, by the AAO-HNS.

15 patients with neurological signs and symptoms, otologic diseases, exposure to noise or tympanic membrane (TM) were taken off the study. Thus, 39 patients with established clinical diagnosis of unilateral or bilateral Meniere’s disease, proven by transtympanic electrocochleography (SP/AP ratio ≥ 35%) were included in the study.

The patients were submitted to full otorhinolaryngological exam, audiometric evaluation and bilateral transtympanic electrocochleography. After these procedures, the patients were divided in 2 groups: Group 1: bilateral Meniere’s disease (electrocochleography with a SP/AP ratio ≥ 35% in both ears) and Group 2: unilateral Meniere’s disease (electrocochleography with a SP/AP ratio ≥ 35% in one single ear).

Initially we carried out a data descriptive analysis. The qualitative variables were presented by means of absolute frequencies (n) and relative frequencies (%). Quantitative variables by mean values, medians, standard deviations, maximum and minimum values.

The two groups were compared by means of the t Student test for the parametric variables (ages). For the non-parametric variables (symptoms progression time and spells’ frequency), the Mann-Whitney and the Fisher’s exact tests were used. The odds ration (OR) was used as an estimate of the relative risk, with a 95% confidence interval. In order to assess possible correlations between symptoms progression times, we used the Spearman correlation coefficient. r values between 0.2 and 0.5 indicating weak correlation, between 0.5 and 0.7, moderate, and greater than 0.8, a strong correlation^27^. The significance level adopted was of 5% (p<0.05).

## RESULTS

Of the 39 patients assessed, 31 were females (79.5%). Patients’ ages when the symptoms started varied between 18 and 72 years, with average age of 42.9 years, with standard deviation of 13.3. Nineteen patients (48.7%) had between 41 and 64 years of age (Table 1).

**Table 1:** Demographic data of 39 patients with definitive diagnosis of Meniere’s disease, proven by transtympanic electrocochleography.

Gender (M/F)	8/31
Age at first consultation*	53.0 13.8
Age of symptoms onset*	42.9 13.3
< 20 years	2 (5.1%)
21- 40 years	16 (41.1%)
41-64 years	19 (48.7%)
>65 years	2 (5.1%)

Legend: * Data expressed in years by means of average standard deviation. M: male and F: female.

Progression time median regarding vertigo was of 7 years, for tinnitus it was of 4 years, for ear fullness 3 years and hearing loss, 5.5 years. Of the 33 patients we had data on, 18 (54.5%) had hearing fluctuation; and of the 35 who had information related to spells periodicity, 23 (65.7%) had frequent spells (daily or weekly). The clinical characteristics of the 39 patients are described on Table 2.

**Table 2:** Auditory and vestibular progression time, expressed in years, hearing fluctuation frequency and daily or weekly spells, expressed in number of episodes, of the 39 patients with a definitive clinical diagnosis of Meniere’s disease.

Vertigo *	7.0 (3.5-15.0)
Tinnitus*	4.0 (2.0-12.0)
Ear fullness*	3.0 (2.0-10.0)
Hearing loss*	5.5 (3.0-12.0)
Hearing fluctuation	18/33
Vertigo spells	23/35

* Data expressed in median and interquartile variation

**Figure 1:**
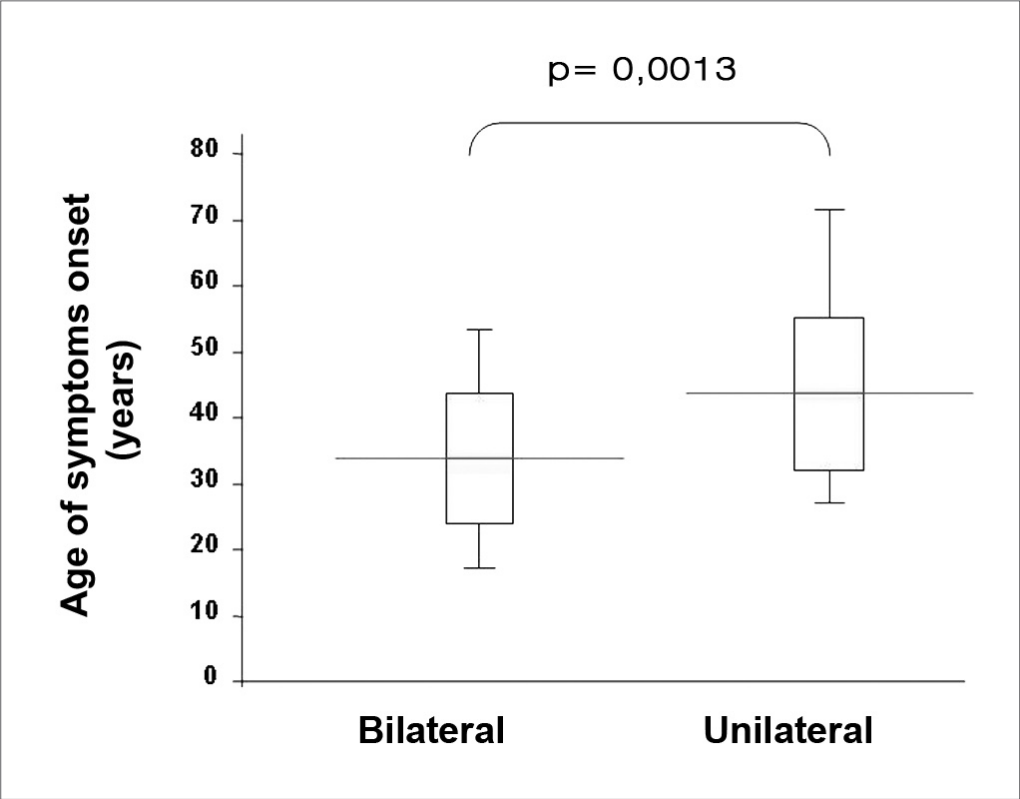
Patients’ ages at symptoms onset, expressed in years, by medians, interquartile variation and extremes, of the 39 patients with definitive clinical diagnosis of Meniere’s disease, with bilateral and unilateral involvement, proven by transtympanic electrocochleography.

Twenty six patients (66.6%) presented with bilateral disease, confirmed by transtympanic electrocochleography. The age-related evaluation at symptoms onset showed that, in the bilateral group, age was lower (33.7 years) than in the unilaterally affected patients (47.5 years) (p= 0.0013, t Student test), according to table 3. By the same token, patients’ age at the time of the fist office visit was also lower in the bilateral group (43.7) when compared to the unilateral group (57.7 years) (p= 0.0018, t Student test) (Figure 1).

**Table 3:** Symptoms onset age and first medical visit, represented in years, of the 39 patients with definitive clinical diagnosis of Meniere’s disease in both, the unilateral and bilateral involvement groups, proven by transtympanic electrocochleography.

	BILATERAL	UNILATERAL	p
N	13	26	
Men/Women	1/12	7/19	0,23
Symptoms onset age*	33.7 11.2	47.5 12.0	0.0013
Age of first medical visit*	43.7 11.1	57.7 12.7	0.0018

Legend: * Data expressed in years by mean standard deviation.

Vertigo, tinnitus and hearing loss evolution time median was slightly lower in the bilateral group, however not reaching statistical significance. There was also no difference between the presence of hearing fluctuation and frequent vertigo spells (daily or weekly) between the groups analyzed (Tables 4 and 5).

**Table 4:** Vestibular and auditory symptoms evolution time, expressed in years of the 39 patients with definitive diagnosis of Meniere’s disease, proven by transtympanic electrocochleography, according to unilateral or bilateral involvement.

	BILATERAL	UNILATERAL	p
N	13	26	
Vertigo**	5.0 (3.0-17.0)	7.5 (4.0-12.0)	0.79
N	12	25	
Tinnitus**	4.0 (1.9-15.3)	5.0 (3.0-10.0)	0.56
N	10	21	
Ear fullness**	3.5 (1.6-12.5)	3.0 (2.0-10.0)	0.85
N	12	25	
Hearing loss**	4.0 (2.0-16.0)	6.0 (3.0-11.5)	0.75

**Table 5:** Hearing fluctuation and vertigo spells, expressed in number of episodes, of the 39 patients with definitive clinical diagnosis of Meniere’s disease, proven by transtympanic electrocochleography, according to unilateral or bilateral involvement.

	BILATERAL	UNILATERAL	p
Hearing fluctuation	5/9	13/24	1,00
Frequency of spells			
Daily	2	7	0,69
Weekly	6	8	0,28
Monthly	1	8	0,21
Annual	2	1	0,23

As far as the disease progression time is concerned, there were no differences in the prevalence of patients with more than 10 years of disease progression between the groups: there were 6 patients (46.1%) in the bilateral group and 11 (42.3%) in the unilateral group, with odds ration (OD) of 1.17 and 95% confidence interval (IC 95%) of 0.31 to 4.46.

There was also no difference between the two groups as far as progression time above 20 years is concerned: 2 patients (15.4%) in the bilateral group and 5 (19.2%) in the unilateral group, with OD= 0.26 [IC 95% 0.03-2.44].

**Figure 2:**
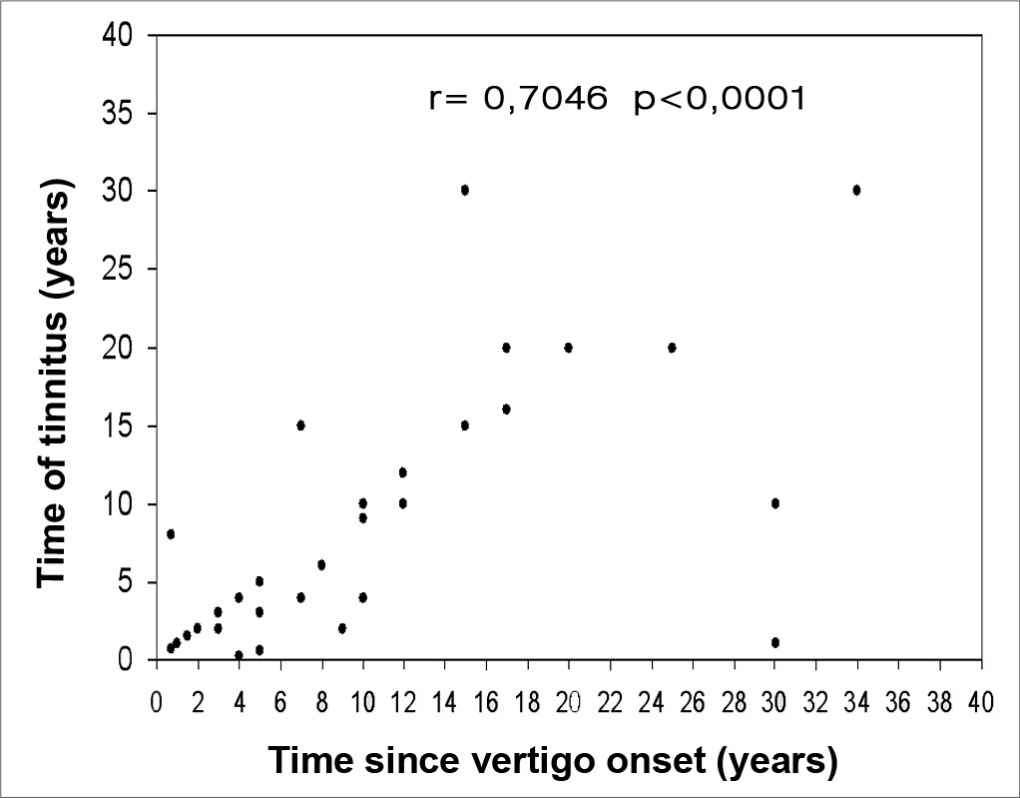
Correlation between tinnitus progression time and vertigo onset, expressed in years, for the 39 patients with definitive clinical diagnosis of Meniere’s disease.

Evaluating the total number of patients, we detected a moderate correlation between disease progression time and the time of tinnitus occurrence (r= 0.7046). By separating the groups we noticed a moderate correlation both in bilateral (r= 0.5115) and in unilateral involvement (r= 0.7715). We observed a weak correlation between disease progression time and ear fullness (r= 0.4699). Alone, we noticed a weak correlation in both groups (r= 0.3945) for the bilateral involvement, and (r= 0.4310) for unilateral. There also was a weak correlation between disease progression time and hearing loss time (r= 0.4859 - Figure 2). By the same token, by separating the groups, we noticed a weak correlation (r= 0.3899) for bilateral involvement, and (r= 0.4753) for unilateral.

## DISCUSSION

In this study we observed that, patients with bilateral involvement have the disease starting earlier on and seek specialized medical advice earlier than those patients with unilateral disease. We did not notice differences in the other clinical variables analyzed (disease progression time, associated symptoms and their characteristics).

A larger prevalence of women (79.5%) was in disagreement with what was seen in previous papers^18^, ^19^, ^20^, ^21^, ^22^, however it was similar to what was seen by Havia et al.^23^ very likely because more women seek doctors when compared to men, in our culture.

We also observed prevalence in individuals on their fourth decades of life, and the average age for symptoms onset was of 42.9 years, similar to what was seen in other studies 23, 24.

Time interval between symptoms onset and specialized treatment was greater in our patients (10.1 years), comparatively to European studies, which vary between 3, 4 and 6 years^23^, ^24^. It is likely that such delay be related to our precarious health care system and the lack of patient education in our country.

Of all the patients studied, more than half had hearing fluctuation and about two thirds complained of frequent vertigo spells (daily or weekly), reflecting its negative impact on life quality.

In this sample, by means of the transtympanic electrocochleography, we detected bilateral involvement in 33.3% of the cases. Other studies showed prevalences of 35%25 and 53%24 of bilateral cases by means of transtympanic electrocochleography. These values may vary according to sample size, hearing loss level, disease progression time and the patient’s clinical complaints on the day of the exam.

It is a consensus among many authors that, the longer the disease progression time, the greater is the percentage of bilateral involvement^18^, ^22^, ^23^, ^24^. We did not observe associations between disease progression time and bilateral involvement, possibly because of the small size of our sample.

According to Havia et al., 44% of the patients with progression times above 20 years had bilateral involvement^23^. In our study, 7 patients had had symptoms for more than 20 years, two of them with bilateral alteration seen at the transtympanic electrocochleography. The small sample size makes these comparisons very limited.

In Meniere’s disease, vestibular symptoms are not necessarily related to cochlear symptoms 20, 26. In fact, we noticed a weak intensity correlation between vertigo progression time and hearing loss.

## CONCLUSION

Most individuals with Meniere’s disease presented symptoms onset after the fourth decade of life. Individuals with bilateral involvement presented symptoms earlier on when compared to those individuals with unilateral symptoms. There were no differences between the bilateral and unilateral groups in relation to disease progression and associated symptoms.
